# Oral health–related quality of life (OHRQOL) of preschool children’s anterior teeth restored with zirconia crowns versus resin-bonded composite strip crowns: a 12-month prospective clinical trial

**DOI:** 10.1007/s00784-021-04359-9

**Published:** 2022-01-06

**Authors:** Ahmad Abdel Hamid Elheeny, Mahmoud Ahmed Abdelmotelb

**Affiliations:** Faculty of Dentistry, Minia University, Ard Shalaby, El Minia, 61519 Egypt

**Keywords:** Child, Quality of life, Oral health, Crowns, Zirconium

## Abstract

**Objectives:**

To compare oral health–related quality of Life (OHRQOL) of preschool children’s anterior teeth restored with prefabricated zirconia crowns (ZC) versus resin-bonded composite strip crown (RCSC).

**Materials and methods:**

A prospective clinical trial included 136 children with early childhood caries aged 36–71 months who were assigned into prefabricated ZC and RCSC groups. A total of 344 teeth were restored either with 170 ZCs (49.4%) and 174 RCSCs (50.6%). Wilson and Cleary’s conceptual model was to associate the study predictors to the OHRQOL. Early Childhood Oral Health Impact Scale (ECOHIS) was used to assess the OHRQOL at 6 and 12 months. Mann–Whitney U test was used in comparing OHRQOL mean scores in the two groups and Wilcoxon signed-rank test with the effect size (*r*) to measure the intragroup OHRQOL change. A Poisson regression model was used to study potential risk factors associated with the overall OHRQOL.

**Results:**

After 12 months, the USPHS parameters of the ZC were significantly superior compared to the RCSC. Overall ECOHIS mean scores in the ZC group were significantly lower than that of the RCSC group at T_1_ and T_2_ (*p* < 0.001). Remarkable enhancement of the OHRQOL at the follow-ups with a large effect size (*r* < 0.8) was observed. Restoration type, retention, baseline OHRQOL, and color had a significant impact on the overall OHRQOL at 12 months.

**Conclusions:**

Preschool children OHRQOL treated with ZC were significantly better than those who received RCSC.

**Clinical relevance:**

One of the optimum treatment standards in pediatric dentistry is the esthetic demand which has significance on the child’s OHRQOL and subsequently child’s general health quality of life. It is beneficial to the dentist to identify the influence of esthetic restorations on the OHRQOL of preschool children which aids in future decision-making. The longitudinal nature of the study enables the dentist to identify the changes of children’s OHRQOL.

**Supplementary Information:**

The online version contains supplementary material available at 10.1007/s00784-021-04359-9.

## Introduction

 Early childhood caries (ECC) refers to the involvement of at least one surface or more of the primary teeth of children below the age of six with a cavitated or non-cavitated carious lesion or missing and/or filled because of caries [1]. Neglect preventive and/or definitive intervention of children suffering from ECC may have led to distressing impacts. For instance, it increases the risk of acquiring a new carious lesion in primary and permanent dentition, hospitalization and increases treatment expenditure and missed school days which subsequently negatively affect the educational attainment and undermine the child’s oral health-related quality of life (OHRQOL) [1, 2]. Untreated carious lesions negatively influenced the multidimensional nature of OHRQOL in terms of oral/dental pain, eating and sleeping difficulties, and diminished psychological and self-image/social activities [3, 4].

To measure the OHRQOL of preschool children, a proxy instrument called Early Childhood Oral Health Impact Scale (ECOHIS) was introduced in 2007 by Pahel et al. [5]. ECOHIS is a sensitive and valid instrument designed to subjectively assess the oral/dental diseases of young children through parental responses to 13 items. The tool items cover different varieties of health-related concepts and their impact on the child and their families [6]. A considerable number of studies adopted ECOHIS to assess the OHRQOL among preschool children. These studies are unanimously agreed that inferior OHRQOL is linked to the untreated ECC.

Various treatment modalities were available to restore anterior teeth affected by ECC. With the increasing parental attention to the esthetic aspect of their children, it is the responsibility of the dentist to choose the suitable restoration, taking into account many considerations. One of these key factors is the parental and children’s perception and satisfaction toward their oral health status. Other significant concerns include the risk of caries, behavior management challenges, and financial issues [7]. It is a hard task to determine the superiority of one coronal restoration of anterior teeth over another.

Different restorations are suggested, for example, Preveneered stainless steel crowns (PVSSCs) which manifest color and surface roughness changes over time and resin facing partial or total detachment [8, 9]. Resin-bonded composite strip crown (RCSC) is a commonly used coronal restoration of the deciduous anterior teeth. The advantages of RCSC are the good esthetic properties and the ability to compensate for the chipped or fractured portions of the composite resin. Regarding RCSC retention rely principally on the existence of an adequate amount of tooth structure and the rigor isolation precautions to avert moisture and/or blood contamination [10]. The RCSC longevity was evaluated in few studies: 2 retrospective studies reported a success rate of 80 percent [10, 11]. Prefabricated zirconia crown (ZC) used in pediatric has become more widely used. The longevity of the prefabricated ZCs was assessed in two previous articles: one study reported a durability of 100 percent [12], and the other reported 98.3 percent after a follow-up period of 12 months [13].

Most of the literature concerned with preschool children is concerned with esthetic restorations, the clinical performance of the deciduous anterior teeth or parental satisfaction toward the restoration. The available data showed no previous trials concerned to compare the OHRQOL before and after restoration of maxillary anterior teeth with prefabricated ZC to RCSC. Hence, the current longitudinal study was conducted to fulfill the following: the primary outcome was to assess the OHRQOL of preschool children who suffered from ECC with their maxillary anterior teeth restored with prefabricated ZC compared to those restored with RCSC. The secondary outcome was to determine the potential risk factors that may be associated with overall OHRQOL.

## Materials and methods

### Ethical approvals

The study was approved by the Ethics Committee of the local Dental School (reference #155/2018) and registered on the ClinicalTrials.gov database (reference #NCT04973761).

### Design and sample size estimation

The study is a prospective parallel randomized clinical trial designed to collect the data on 3 occasions: at the baseline (i.e., preoperatively) (T_0_), at 6 months (T_1_) and 12 months (T_2_). To estimate the sample size, a general linear mixed model power and sample size (GLMMPSS) (URL http://glimmpse.samplesizeshop.org/) was used [14]. The required sample size with a statistical power of 90% was 120 children. After adding 15% to compensate for drop-off, the total sample size was 140 children. Based on the mean and standard deviation (SD) of repeated measures of 14 children included in a pilot study, the following inputs were specified: (i) primary hypothesis was treatment-by-time interaction using Hotelling-Lawley Trace statistical test; (ii) for ZC, the total OHRQOL means at T_0_ = 17.22; T_1_ = 8.84; and T_2_ = 8.96; and (iii) for RCSC, the total OHRQOL means at T_0_ = 18.27; T_1_ = 9.08, and T_2_ = 9.31, (iv) SD (constant) = 0.25. The type 1 error cut-off was 0.05.

### Setting, randomization, and allocation

**Fig. 1:**
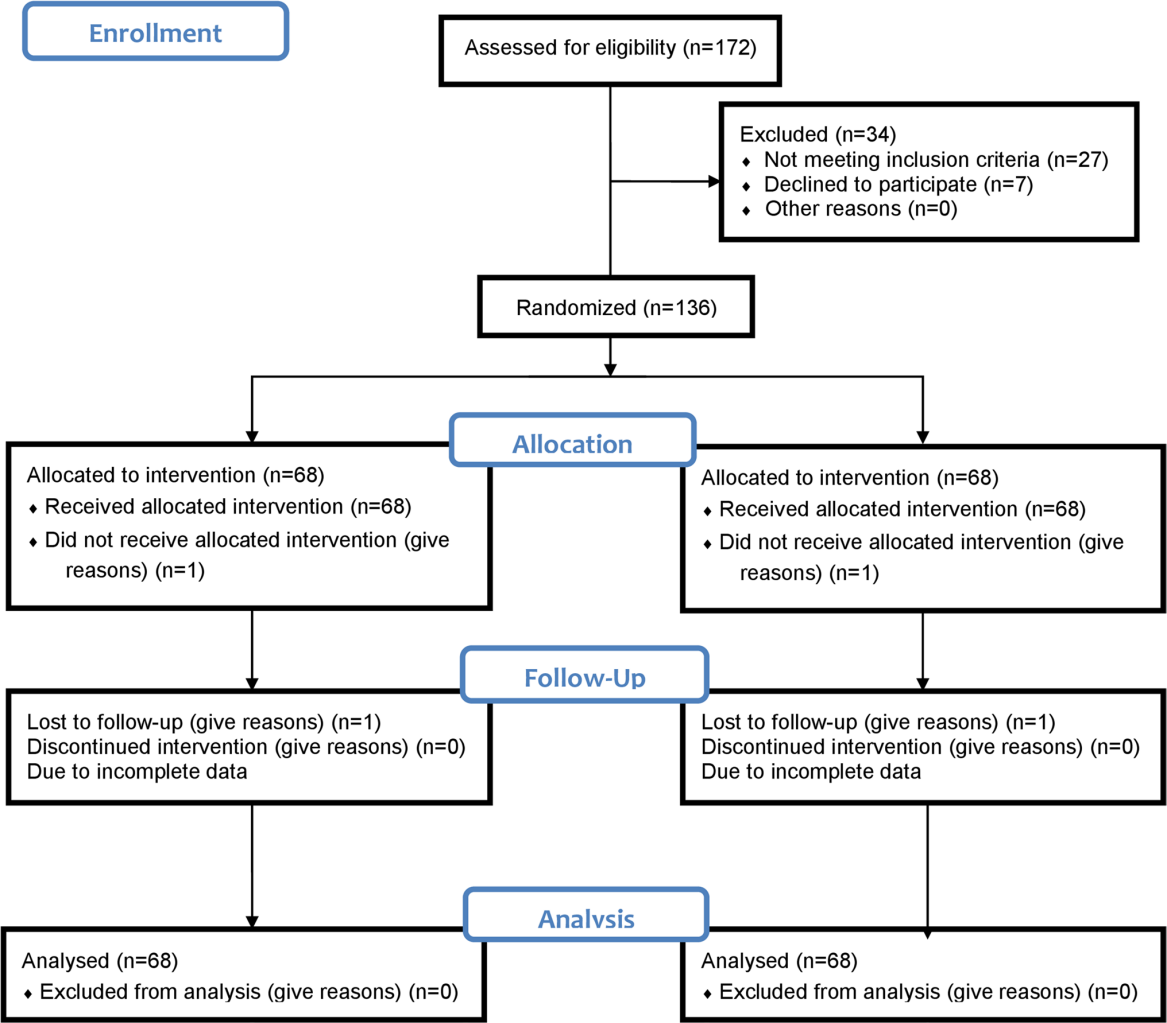
Consort Flowchart of the trial design

Children were voluntarily included from those attending the outpatient clinic of Pediatric Dentistry Department. The trial was started in March 2018 to November 2020. Recruited children were randomly assigned into two equal groups (69 per group) using a block randomization software (block of 4) https://www.sealedenvelope.com/simple-randomiser/v1/lists (Fig. 1). Tightly sealed opaque envelopes which were included the restoration type were randomly allocated to each participant and opened at the time of treatment. The random allocation sequence was performed by an independent researcher (E.K.M) [15, 16]. The statistician was blinded to the type of restoration during data analysis. In group “1,” teeth were restored prefabricated primary ZCs (NuSmile Ltd., Houston, Texas, United States). Teeth in group “2” were restored with RCSC (3 M™ Strip Crown Form, ESPE, Dental Product).

### Recruitment standards

Eligible children aged from 36 to 71 months with at least 2 maxillary anterior teeth with extensive cervical decalcification or at least the presence of two carious surfaces according to the American Academy of Pediatric Dentistry indications for full coronal restorations [13, 17]. Based on Wyne nomenclature for ECC, children with type I (mild/moderate type usually involves 2 maxillary incisors) or type II (moderate/sever type involves labiolingual carious lesions affecting maxillary incisors, with or without molar caries while the mandibular incisors that remain unaffected were enrolled [18]. Regarding the progression of ECC lesion, teeth in circular stage (i.e., lesion in the dentin and circular distribution of this lesion proximally) or in destructive stage (i.e., destruction of more than half the crown without affecting the incisal edge) [19]. Children should have no previous dental experience. Teeth should be vital and need restorative dentistry or vital pulp therapy. Caries could be extensive but confined to one surface or moderate included 2 surfaces [20]. Preoperatively, all teeth were checked radiographically and those indicated for pulpotomy were included according to the AAPD criteria. Pulpotomy was indicated upon carious pulp exposure with normal or with signs of reversible pulpitis that was confirmed radiographically (i.e., negative radiographic findings of periapical radiolucency or pathologic resorption). After exacerbation of the coronal pulp tissues, the radicular pulp tissues must be vital without signs of necrosis, suppuration, or excessive uncontrolled bleeding by a cotton pellet applied for several minutes [21–23]. Included children should be cooperative rated number 3 or 4 according to the Frankl behavior rating scale and categorized as class 1 or 2 according to the American Society of Anesthesiologists (ASA) classification. Children with non-restorable teeth, severe intellectual, emotional, or obvious behavior problems were precluded from the study [15].

### Theoretical model and data acquisition

**Fig. 2:**
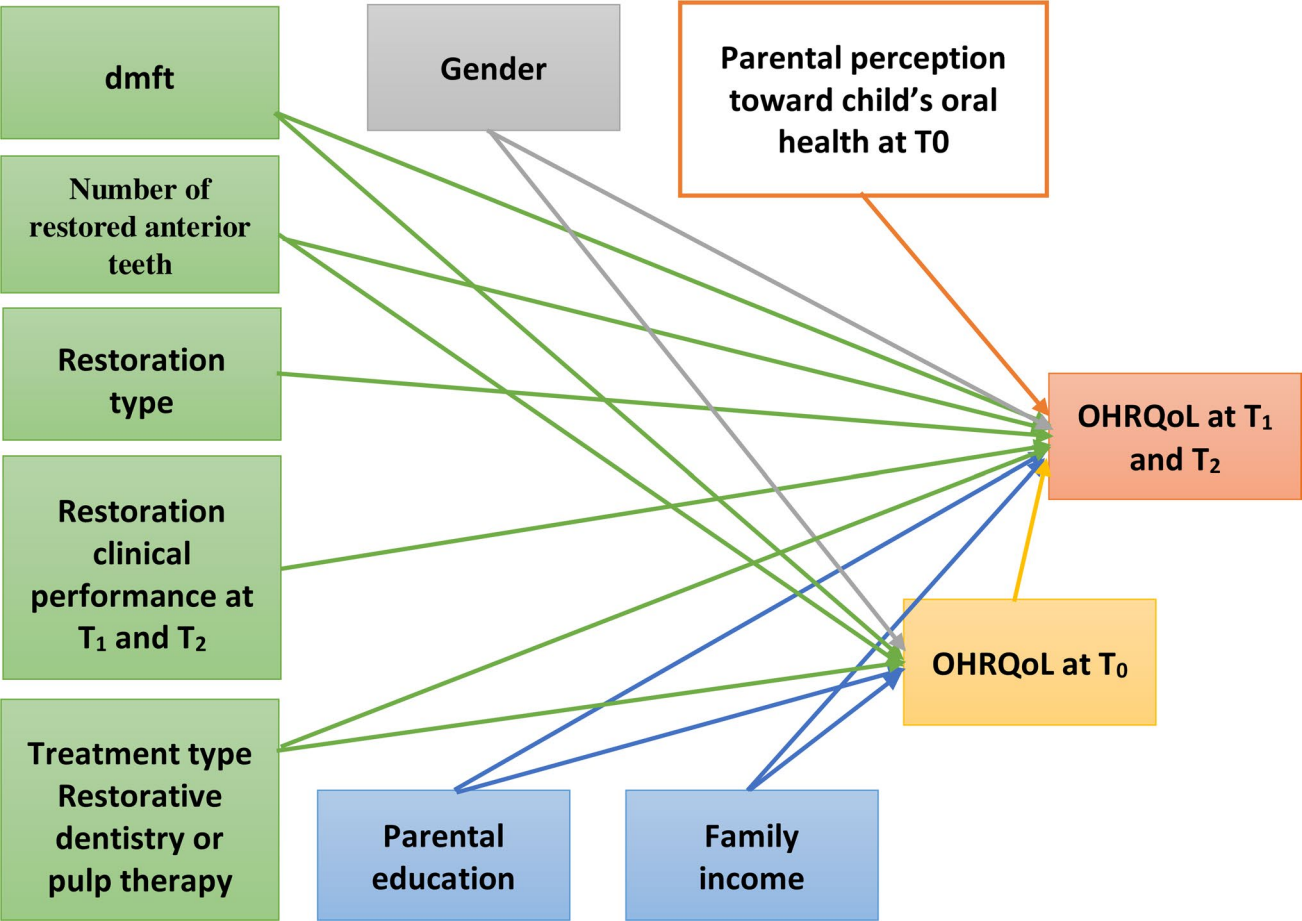
Wilson and Cleary regression model of the proposed predictors of the overall oral health related quality of life at 6 and 12 months (OHRQOL)

Before starting the clinical procedures, the chief investigator (E.A.A) collected the data from the parents/caregivers of the enrolled children via a self-administrated questionnaire. According to Wilson and Cleary’s theoretical model, the association between the OHRQOL and the independent predictors was handled as presented in Fig. 2. The independent variables at T_0_ included the demographic data (Gender and age in months), parental socioeconomic status (SES) included 2 items: (i) mother and father schooling which classified into high (higher than secondary school), intermediate (secondary school), and low (less than secondary school or illiterate); (ii) household expenditure was categorized according to the annual income of the average families in the local currency which is equivalent to USD into two classes: < 300 USD and ≥ 300 USD per month. Caries experience was assessed using decayed, missing, filled teeth (dmft) index. The scores of dmft were dichotomized into < 3 and ≥ 3. The number of anterior teeth needs restoration that dichotomized into 2 teeth or > 2 teeth. A global question regarding the parental psychosocial variable “How do you rate your child’s oral health?” five responses rated from 0 to 4 on the Likert’s points scale (Poor = 0, Fair = 1, Average = 2, Good = 3, and Excellent = 4). The parental perception was assessed at T_0_, T_1_, and T_2_. At 6 and 12 months, the frequency distribution of restoration type (ZC or RCSC) and assessment was added. Based on the modified United States Public Health Service (USPHS) criteria, failure of restoration types was identified [24]. Restorations were evaluated for (i) the restoration retention categorized into (A) intact, (B) chipped/small but noticeable areas of loss of material, (C) large loss of material, and (D) total loss; (ii) color match categorized into (A) no noticeable difference from adjacent teeth, (B) slight shade mismatch, and (C) obvious shade mismatch, finally; (iii) the restoration contour categorized into (A) crown is cosmetic, natural-looking, size/shape is acceptable, not ideal (B), and (C) Crown not esthetic, detracts from appearance of the mouth. For data analysis, each domain of the restoration assessment was dichotomized into “success” for (A) and (B) scores and “failure for (C) and/or (D) scores. Pulp condition was dichotomized into “success” indicating the absence of any adverse clinical signs or symptoms, such as sensitivity, mobility, pain, or swelling and “failure” indicating the presence of one or more of clinical signs or symptoms. Wear of opposing teeth was scored according to the Smith and Knight tooth wear index criteria [13, 25]: no loss of enamel surface characteristics; no loss of contour (score 0); loss of enamel surface characteristics, minimal loss of contour (score 1); loss of enamel exposing dentine for less than one third of surface, loss of enamel just exposing dentin, defect less than 1-mm deep (score 2); loss of enamel exposing dentin for more than one third of surface, loss of enamel and substantial loss of dentin, defect less than 1–2-mm deep (score 3); Complete enamel loss, pulp exposure, secondary dentin exposure, pulp exposure or exposure of secondary dentin, defect more than 2-mm deep, pulp exposure, and secondary dentin exposure (score 4). To dictomize the findings; score “0” was considered a “success,” while other scores were a “failure.”

### OHRQOL assessment instrument

An Arabic validated version of ECOHIS was used to assess the OHRQOL of the preschool children. ECOHIS encloses 13 questions and each question has 5 responses that were recorded by the parents. Responses are rated from never (score 0), almost never (score 1), occasionally (score 2), often (score 3), very often (score 4), and (score 5) I don’t know. The ECOHIS has 2 sections; (i) the child impact section (CIS) includes 4 domains: *symptom* domain (question #1), *function* domain (questions #2 to #5), *psychological* domain (questions #6 and #7), and s*elf-image/social interaction* domain (questions #8 and #9); (ii) the family impact section (FIS) consists of 2 domains: *family distress* domain (questions #10 and #11) and *family function* domain (questions #12 and #13). For each child, the OHRQOL is calculated by summing the score of the child impact part and the family part independently. For the CIS and FIS, the minimum and maximum total scores are ranged from 0 to 36 and 0 to 16, respectively. The higher ECOHIS score, the poorer OHRQOL and vice versa. Score 5 (i.e., I don’t know) is treated as a missing item. The questionnaire was cancelled if there are more than 2 missing responses in the CIS or one response in the FIS and another child was included. A questionnaire with permitted missing items is assigned as an average of the residual units for that part [5, 26].

### Clinical procedures

Anterior teeth were anesthetized using lidocaine hydrochloride 2% and epinephrine 1:100,000 (Lignospan® standard, 1.7 mL, SEPTODONT Inc.) then isolated with a rubber dam. For the teeth that underwent pulp therapy, caries was removed, access was gained, and the entire pulp chamber roof was removed. Coronal pulp tissue was extirpated with a sharp excavator. The pulp stump was covered with a thick mix of polymer reinforced Zinc-oxide/Eugenol (ZOE) (Zinconol, Prevest DenPro) after a 5-min application of a cotton pellet soaked in formocresol (Sultan, USA). For prefabricated ZC, tooth reduction was performed as follows: (i) A 1.5 mm of the incisal edge was removed using TR-13 and WR-13 diamond burs (Mani, Inc., Japan); (ii) the axil wall was prepared in a circumferential manner by approximately 20–30% and ended from 1 to 2 mm subgingivally into a smooth feather-edged cervical margin according to the NuSmile manufacturers’ technical guidelines. For the hemostasis after subgingival preparation, a pellet soaked in epinephrine 1 mg/ml (Primer Dental Products Company) was maintained over the gingiva for 1 min with gauze pressure. Any residual coagulum was removed using suction or moistened gauze; (iii) pink crown was used to ensure passive fitting with a negative pressure during crown application and the checking of the occlusion on the bite; and finally, (iv) the suitable-sized ZC was cemented using a dual-cured, self-adhesive resin cement (TotalCem, ITENA Clinical Products). Initial curing with LED light cure (1200 mW/cm^2^) (Elipar™, 3 M ESPE) for 3 s then the excess was removed with scaler and super floss before final curing for additional 40 s.

For the RCSC, proper shade and strip crown size were selected using 3 M Filtek Z350 XT shade guide. A venting hole to prevent air bubble trapping within the crown was made mesial to the incisal edge. For the pulpotomized incisors, a layer of glass ionomer (Fuji EQUIA® Fil, GC) was added to avoid the interference with composite resin setting. For the non-exposed teeth, a resin-modified glass ionomer liner/base (Vitrebond™, 3 M ESPE dental products) was placed over exposed dentin for protection. A 37% phosphoric acid-etching gel (3 M Scotchbond™ Etchant) for 15 s was applied then rinsed for 60 s and dried for 30 s with a moisture-free air. Scotchbond light-cured bonding agent (3 M ESPE dental product) was applied over the etched enamel and thinned by a moisture-free air. Each tooth was cured separately. The composite resin (Filtek Z350 XT WD (3 M ESPE Dental Products) was added in increments of thickness of 1.5 mm then light-cured for 20 s using 3 M™ Elipar™ DeepCure-L LED curing light (with output 1000–2000 mW/cm2). The strip crown was removed from the palatal side with an explorer. Finally, occlusal adjustments, finishing, and polishing were performed using diamond burs (KG Sorensen), Sof Lex discs (3 M), and polishing strips. The labial surface was left without polishing to preserve the luster of the labial surface.

### Calibration and reliability

Two independent pediatric dentists (H.O.S and H.M.N) with an experience of 10 years were responsible for the clinical evaluation of the 2 restorations. Clinical assessment was performed separately and inter-observer reliability using Kappa coefficients (κ) and intra-class correlation coefficient (ICC) at T_1_ and T_2_. The values of *κ* were high at the two follow-up episodes (*κ* = 0.92 and 0.89 at T_1_ and T_2_, respectively) and ICC exceeded 0.91 at the two follow-ups.

### Data analysis

Descriptive statistics including independent predictors (i.e., demographic, SES, number of anterior teeth needs restoration, parental perception, dmft, restoration type, and success) and dependent variable (i.e., ECOHIS item scores) were expressed in proportions and means/standard deviation at the baseline (T_0_) and follow-ups (T_1_ and T_2_). To compare ZC and RCSC in relation to the independent predictors, chi-square test was used in comparing observed frequencies. The Kolmogorov–Smirnov and Shapiro–Wilk tests were used to specify the data distribution. As data were not normally distributed, the Mann–Whitney U test was used to determine if there was a statistically significant difference between ZC and RCSC means of different ECOHIS domains. For each restoration type, the ECOHIS domain mean changes between the baseline (T_0_) and follow-ups (T_1_) and (T_2_) and the mean difference between T_1_ and T_2_ to measure the intra-group OHRQOL improvement was analyzed using Wilcoxon signed-rank test. The non-parametric effect size (*r*) of the mean differences was calculated by dividing the absolute standardized statistic (z) of the Wilcoxon signed-rank test by the square root of the pair number (z/√N). Interpreting the “*r*” values was as follows: small effect size (***r*** < 0.3), moderate effect size (*r* = 0.3–0.8), and large effect size (*r* > 0.8) [27].

To study the impact of the independent predictors and the baseline OHRQOL scores (T_0_) on the overall OHRQOL score (i.e., total ECOHIS scores) at the end of follow-up (T_2_), univariate and multiple Poisson regression analysis with robust variance was used. Independent variables with a statistical significance of < 0.2 were included into the final adjusted multivariate model analysis exclusively. Relative risk (RR) was calculated to compare the effect measure at 95% *CI*. All statistical tests were conducted assuming a level of significance of 5%. Data were analyzed based on the Statistical Program Statistical Package for the Social Sciences (SPSS software version 22, IBM Corp., Armonk, NY).

## Results

Of 172 children examined for recruitment eligibility, 138 children were included (36 children were excluded for different reasons that were explained in Fig. 1). A high response rate of 98.55% was reported. Only 2 cases (one of each group) were failed to attain at T_1_, so their records were cancelled and not incorporated in the final analysis.

Table 1 shows the independent predictors (demographic, socioeconomic, parental perception toward their children’s oral health, clinical status in terms of dmft) plus the restoration type, and clinical success at 6 and 12 months. At T_2_, parents were more satisfied with the ZC over the RCSC (*p* = 0.02). Also, the ZC showed a higher retention rate over the RCSC (*p* = 0.002). While two children treated with the RCSCs showed total loss of the crowns, and 7 subjects suffered from partial loss of the composite resin restoration considered (13.2%). Children with ZC showed a significantly better color match and contour than those that received RCSC at T_2_ (*p* = 0.005).

**Table 1:** Demographic, socioeconomic, and parental perception toward their children oral health and clinical status of ZC and RCSC

Predictors	ZC *N* (%) *N* = 68	RCSC *N* (%) *N* = 68	P*
Gender			
Girls	38 (55.9)	36 (52.9)	0.30
Boys	30 (44.1)	32 (47.1)	
Age (years)			
3–4	29 (42.6)	30 (44.1)	0.86
5–5.92	39 (57.4)	38 (55.9)	
Number of restored anterior teeth			
= 2	30 (44.1)	35 (51.5)	0.39
> 2	38 (55.9)	33 (48.5)	
Treatment type			
Restorative dentistry	26 (38.2)	29 (42.6)	0.73
Pulp therapy	42 (61.8)	39 (57.4)	
Mother schooling			
High	52 (76.5)	55 (80.9)	0.12
Intermediate	13 (19.1)	7 (10.3))	
Low	3 (4.4)	6 (8.8)	
Father schooling			
High	46 (67.7	49 (72.1)	0.53
Intermediate	13 (19.1)	14 (20.6)	
Low	9 (13.2)	5 (7.4)	
Family income per month			
< 300 USD	33 (48.5)	31 (45.6)	0.49
≥ 300 USD	35 (51.5)	37 (54.4)	
dmft			
≥ 3	40 (58.8)	44 (64.7)	0.36
< 3	28 (41.2)	24 (25.8)	
Parental perception toward his/her child’s oral health			
T_0_			
Poor/fair	59 (86.8)	60 (88.2)	0.80
Average	9 (13.2)	8 (11.8)	
Good/excellent	0 (0)	0 (0)	
T_1_			
Poor/fair	0 0)	2 (2.9)	0.22
Average	10 (14.7)	14 (20.6)	
Good/excellent	58 (85.3)	52 (76.5)	
T_**2**_			
Poor/fair	2 (2.9)	10 (14.7)	**0.02**
Average	9 (13.2)	14 (20.6)	
Good/excellent	57 (83.8)	44 (64.7)	
Restoration assessment 1. Retention			
T_1_			
Success	67 (98.5)	66 (97.1)	0.56
Failure	1 (1.5)	2 (2.9)	
T_2_			
Success	67 (98.5)	59 (86.8)	**0.01**
Failure	1 (1.5)	9 (13.2)	
2. Color			
T_1_			
Success	67 (98.5)	63 (92.6)	0.09
Failure	1 (1.5)	5 (7.4)	
T_2_			
Success	67 (98.5)	58 (85.3)	**0.005**
Failure	1 (1.5)	10 (14.7)	
3. Contour			
T_1_			
Success	67 (98.5)	65 (95.6)	0.31
Failure	1 (1.5)	3 (4.4)	
T_2_			
Success	67 (98.5)	58 (85.3)	**0.005**
Failure	1 (1.5)	10 (14.7)	
Pulp condition			
T_1_			
Success	67 (98.5)	68 (100)	0.32
Failure	1 (1.5)	0 (0)	
T_2_			
Success	67 (98.5)	68 (100)	0.32
Failure	1 (1.5)	0 (0)	
Wear of opposing teeth			
T_1_			
Success	67 (98.5)	68 (100)	0.32
Failure	1 (1.5)	0 (0)	
T_2_			
Success	62 (91.2)	68 (100)	**0.03**
Failure	6 (8.8)	0 (0)	

P*: chi-square test; *p*-value was set to 0.05

T0, baseline; T1, first follow-up period at 6-month; T_2_, second follow-up period at 12-month

*ECOHIS*, early childhood oral health impact scale

*ZC*, zirconia crown; *RCSC*, resin-bonded composite strip crown; *dmft*, decayed, missing, filled teeth

Data in Table 2 displays the frequency distribution of pulpotomized and non-pulpotomized teeth, modified, USPHS criteria for each tooth, and wear of opposing teeth of ZC and RCSC. A total of 344 teeth were restored either with 170 ZCs (49.4%) or 174 RCSCs (50.6%). For the ZC group, 140 teeth were pulpotomized (82.4%) and 30 teeth were non-pulptomized (17.6%). For the RSCS group, 148 were pulpotomized (85.1%) and 26 teeth were non-pulpotomized (14.9%). At T_1_, no significant difference between both restorations was found regarding the USPHS parameters and wear of opposing teeth, while at T_2_, the USPHS parameters of the ZC were significantly superior compared to the RCSC. In contrast, the wear of opposing teeth was significantly higher among the ZC group (*p* < 0.01). Only one child with two teeth restored with ZCs (1.2%) suffered from chronic abscess with a fistulous tract formation after 6-month follow-up. The difference between the two groups was non-significant at T_1_ and T_2_.

**Table 2:** Frequency distribution of pulpotomized/non-pulpotomized teeth, restoration clinical assessment, and wear of opposing teeth of ZC and RCSC

Predictors	ZC *N* (%) *N* = 170	RCSC *N* (%) *N* = 174	P*
Pulpotomized	140 (82.4)	148 (85.1)	
Non-pulpotomized	30 (17.6)	26 (14.9)	0.56
Restoration assessment 1. Retention			
T_1_			
Success	168 (98.8)	170 (97.7)	0.43
Failure	2 (1.2)	4 (2.3)	
T_2_			
Success	168 (98.8)	156 (89.7)	** < 0.001**
Failure	2 (1.2)	18 (10.3)	
2. Color			
T_1_			
Success	166 (97.6)	164 (94.3)	0.11
Failure	4 (2.4)	10 (5.7)	
T_2_			
Success	166 (97.6)	156 (89.7)	0.002
Failure	4 (2.4)	18 (10.3)	
3. Contour			
T_1_			
Success	168 (98.8)	168 (96.6)	0.16
Failure	2 (1.2)	6 (3.4)	
T_2_			
Success	168 (98.8)	154 (88.5)	** < .001**
Failure	2 (1.2)	20 (11.5)	
Pulp condition			
T_1_			
Success	168(98.8)	174(100)	0.15
Failure	2(1.2)	0(0)	
T_2_			
Success	168 (98.8)	174 (100)	0.15
Failure	2 (1.2)	0 (0)	
Wear of opposing teeth			
T_1_			
Success	170 (100)	174 (100)	1
Failure	0 (0)	0 (0)	
T_2_			
Success	160 (94.1)	174 (100)	** < 0.001**
Failure	10 (5.9)	0 (0)	

*Chi-square test; *p*-value was set to 0.05

T0, baseline; T1, first follow-up period at 6-month; T_2_, second follow-up period at 12-month

*ZC*, zirconia crown; *RCSC*, resin-bonded composite strip crown; *dmft*, decayed, missing, filled teeth

At T_0_, all ECOHIS items showed no significant difference which was found in relation to the restoration type. At T_1_ and T_2_, the frequency of toothache was significantly decreased without a significant difference between the two restorations. At T_1_ and T_2_, the frequency of the items concerned with the child’s self-image/social interaction (smiling and talking) and family distress (feeling upset and guilt) of children treated with ZC was significantly better than those of the other group (Table 3).

**Table 3:** ECOHIS rating scores frequency distribution of ZC and RCSC at T_0_, T_1_ and T_2_

ECOHIS questions	T_0_		T_1_		T_2_	
	ZC	RCSC	ZC	RCSC	ZC	RCSC
Q1. Had pain in the teeth, mouth or jaws?						
Never/almost never	10(14.7)	8(11.8)	66(97.1)	68(100)	68(100)	68(100)
Occasionally	9(13.2)	2(2.9)	2(2.9)	0(0)	0(0)	0(0)
Often/very often	49(72.1)	58(85.3)	0(0)	0(0)	0(0)	0(0)
P*	0.07		N/A		N/A	
Q2. Had difficulty drinking hot or cold beverages?						
Never/almost never	0(0)	5(7.4)	68(100)	68(100)	68(100)	68(100)
Occasionally	23(32.8)	17(25)	0(0)	0(0)	0(0)	0(0)
Often/very often	45(66.2)	46(67.6)	0(0)	0(0)	0(0)	0(0)
P*	0.06		N/A		N/A	
Q3. Had difficulty to chew food?						
Never/Almost never	5(7.4)	12(17.6)	68(100)	68(100)	68(100)	66(88.2)
Occasionally	23(33.8)	17(25)	0(0)	0(0)	0(0)	28.8)
Often/very often	40(58.8)	39(57.4)	0(0)	0(0)	0(0)	0(0)
P*	0.15		N/A		0.08	
Q4. Had difficulty for pronouncing any words?						
Never/Almost never	53(78)	57(83.8)	66(97.1)	62(91.2)	66(97.1)	60(79.4)
Occasionally	12(17.6)	9(13.2)	2 (2.9)	5(7.4)	2 (2.9)	6(17.6)
Often/very often	3(4.4)	2(2.9)	0(0)	1(1.5)	0(0)	2(2.9)
P*	0.68		0.30		0.12	
Q5. Missed pre-school or day-care?						
Never/almost never	29(42.6)	23(33.8)	62(91.2)	62(91.2)	68(100)	68(100)
Occasionally	9(13.2)	9(13.2)	6(8.8)	6(8.8)	0(0)	0(0)
Often/very often	30(44.2)	36(53)	0(0)	0(0)	0(0)	0(0)
P*	0.54		N/A		N/A	
Q6. Had difficulty sleeping?						
Never/almost never	28(41.1)	26(38.2)	66(97.1)	68(100)	68(100)	66(97.1)
Occasionally	13(19.1)	11(16.2)	2 (2.9)	0(0)	0(0)	2(2.9)
Often/very often	27(39.8)	31(45.6)	0(0)	0(0)	0(0)	0(0)
P*	0.77		0.15		0.77	
Q7. Been annoyed or bad-tempered?						
Never/almost never	41(60.4)	41(60.2)	68(100)	65(95.6)	68(100)	65(95.6)
Occasionally	5(7.4)	5(7.4)	0(0)	2(2.9)	0(0)	3(4.4)
Often/very often	22(32.4)	22(32.4)	0(0)	1(1.5)	0(0)	0(0)
P*	N/A		0.22		0.08	
Q8. Avoided laughing or smiling when around other children?						
Never/almost never	27(39.8)	24(35.3)	68(100)	57(83.8)	68(100)	55(80.9)
Occasionally	18(26.4)	16(23.5)	0(0)	6(8.8)	0(0)	8(11.4)
Often/very often	23(33.8)	28(41.2)	0(0)	5(7.4)	0(0)	5(7.4)
P*	0.68		**0.003**		**0.001**	
Q9. Avoided talking?						
Never/almost never	31(54.5)	30(44.1)	67(98.5)	60(88.2)	67(98.5)	51(75)
Occasionally	29(42.6)	30(44.1)	1(1.5)	7(10.3)	0(0)	16(23.5)
Often/very often	8(11.8)	8(11.8)	0(0)	1(1.5)	1(1.5)	1(1.5)
P*	0.98		**0.03**		** < 0.001**	
Q10. Felt upset?						
Never/almost never	5(7.4)	8(11.8)	66(97.1)	55(80.9)	68(100)	55(80.9)
Occasionally	23(33.8)	23(33.8)	2 (2.9)	3(4.4)	0(0)	2(2.9)
Often/very often	40(58.8)	37(54.4)	0(0)	10(14.7)	0(0)	11(16.2)
P*	0.81		**0.004**		**0.001**	
Q11. Felt guilty?						
Never/almost never	10(14.7)	15(22.1)	67(98.5)	59(86.8)	67(98.5)	55(80.9)
Occasionally	17(25)	11(16.2)	0(0)	4(5.9)	0(0)	2(2.9)
Often/very often	41(60.3)	42(61.8)	1(1.5)	5(7.4)	1(1.5)	11(16.2)
P*	0.32		**0.03**		**0.003**	
Q12. Had to take hours or days off work?						
Never/almost never Occasionally	12(17.6) 19(27.9)	10(14.7) 24(35.3)	68(100) 0(0)	67(98.5) 1(1.5)	67(98.5) 0(0)	67(98.5) 1(1.5)
Often/very often	37(54.4)	34(50)	0(0)	0(0)	1(1.5)	0(0)
P*	0.64		0.32		0.37	
Q13. Had the family’s economic situation affected?						
Never/almost never	35(51.5)	39(57.4)	35(51.5)	36(52.9)	35(51.5)	38(55.9)
Occasionally	16(23.5)	19(27.9)	25(36.8)	22(32.4)	25(36.8)	20(29.4)
Often/very often	17(25)	10(14.7)	8(11.8)	10(14.7)	8(11.8)	10(14.7)
P*	0.32		0.50		0.50	

P*, chi-square test; *p*-value was set to 0.05

T_0_, baseline; T_1_, first follow-up period at 6-month; T_2_, second follow-up period at 12-month

*ECOHIS*, early childhood oral health impact scale

ZC, zirconia crown; *RCSC*: resin-bonded composite strip crown

**Fig. 3:**
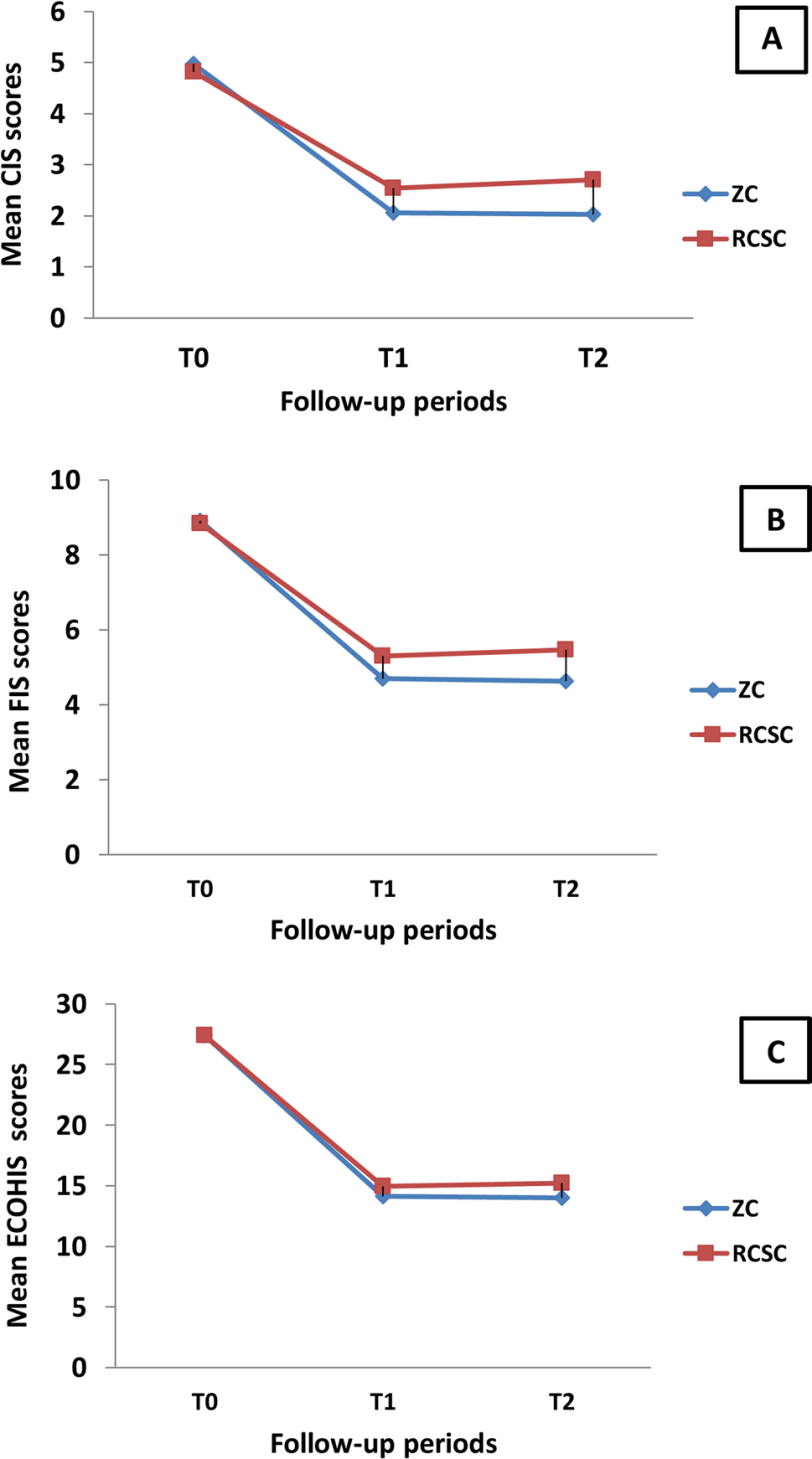
Child impact score (CIS) overall mean scores (A), family impact score overall mean scores (FIS), and early childhood oral health impact scale (ECOHIS) overall mean scores of zirconia crown (ZC) and resin-bonded composite strip crown at 6 months (T1) and 12 months (T2)

Table 4 and Fig. 3 show that the overall CIS and overall ECOHIS mean scores of the ZC group were significantly lower than that of the RCSC group at T_1_ and T_2_ (*p* < 0.001) indicating a better OHRQOL of children treated with ZC. Only the family distress domain of the ZC group was significantly less than that of the RCSC group at the follow-ups (*p* < 0.001).

**Table 4:** Mean and median of ECOHIS domains of ZC and RCSC at T_0_, T_1_, and T_2_

ECOHIS domains	ZC		RCSC		P*
	Mean ± SD	Median (IQR)	Mean ± SD	Median (IQR)	
Child symptoms					
T_0_	2.57 ± 0.74	3 (1)	2.74 ± 0.66	3 (1)	0.09
T_1_	1	1 (0)	1	1 (0)	N/A
T_2_	1	1 (0)	1	1 (0)	N/A
Child function					
T_0_	8.46 ± 1.79	9 (6)	8.38 ± 1.66	8 (7)	0.77
T_1_	4.09 ± 0.37	4 (2)	4.24 ± 0.66	4 (2)	0.10
T_2_	4.03 ± 0.17	4 (1)	4.31 ± 0.53	4 (2)	** < 0.001**
Child psychology					
T_0_	3.71 ± 1.76	3 (4)	3.79 ± 1.64	3.5 (4)	0.64
T_1_	2.03 ± 0.17	2 (1)	2.06 ± 0.93	2 (2)	0.64
T_2_	2	2 (0)	2.07 ± 0.62	2 (1)	**0.02**
Child self-image and social interaction					
T_0_	3.60 ± 1.49	4 (4)	3.74 ± 1.43	4 (4)	0.52
T_1_	2.03 ± 0.24	2 (2)	2.37 ± 0.64	2 (2)	** < 0.001**
T_2_	2.05 ± 0.24	2 (2)	2.53 ± 0.76	2 (1)	** < 0.001**
Overall CIS					
T_0_	18.34 ± 3.01	19 (13)	18.65 ± 2.82	18 (14)	0.61
T_1_	9.15 ± 0.55	9 (3)	9.67 ± 1.04	9 (4)	** < 0.001**
T_2_	9.08 ± 0.29	9 (2)	9.91 ± 1.06	10 (3)	** < 0.001**
Family distress					
T_0_	4.97 ± 1.05	6 (4)	4.82 ± 1.44	6 (4)	0.93
T_1_	2.06 ± 0.29	2 (2)	2.54 ± 0.85	2 (2)	** < 0.001**
T_2_	2.03 ± 0.24	2 (2)	2.71 ± 0.96	2 (3)	** < 0.001**
Family function					
T_0_	4.10 ± 1.26	4 (4)	4.01 ± 1.23	4 (4)	0.60
T_1_	2.63 ± 0.73	3 (2)	2.76 ± 0.83	2 (2)	0.13
T_2_	2.60 ± 0.74	3 (2)	2.75 ± 0.84	2 (2)	0.06
Overall FIS					
T_0_	8.91 ± 2.05	9 (8)	8.83 ± 2.23	7 (7)	0.34
T_1_	4.69 ± 0.93	5 (2)	5.30 ± 1.18	5 (4)	0.36
T_2_	4.63 ± 0.99	5 (4)	5.46 ± 1.28	5.5 (4)	0.06
Overall ECOHIS score					
T_0_	27.41 ± 5.60	31 (19)	27.40 ± 4.38	29 (19)	0.92
T_1_	14.12 ± 0.97	14 (4)	14.94 ± 1.67	15 (6)	**0.006**
T_2_	14 ± 1.01	14 (4)	15.22 ± 1.74	15 (6)	** < 0.001**

P*, Mann–Whitney U test; *p*-value was set to 0.05

*SD*, standard deviation; *IQR*: inter-quartile range

T_0_, baseline; T_1_, first follow-up period at 6-month; T_2_, second follow-up period at 12-month

*ECOHIS*, early childhood oral health impact scale; *CIS*, child impact scale; *FIS*, family impact scale

*ZC*, zirconia crown; *RCSC*, resin-bonded composite strip crown

The overall mean difference before and after treatment at 6 and 12 months (T_1_ − T_0_ and T_2_ − T_0_) was statically significant, with a large effect size (*r* < 0.8; *p* < 0.001) denoting an outstanding enhancement of the OHRQOL in the two groups. All domains in both groups showed a large effect size except for the child psychology and child self-image/social interaction domains, which was moderate. The magnitude of difference between the first and second follow-up occasions for the overall CIS, FIS, and ECOHIS was small (i.e., small effect size) in the two groups (*p* > 0.05) (Table 5).

**Table 5:** Effect size (*r*) and change in the mean of ECOHIS domains between the baseline and follow-ups (T_1–_T_0_ and T_2–_T_0)_ and between the first and second follow-ups (T_2–_T_1_) of both restorations (ZC and RCSC)

ECOHIS domains	ZC			RCSC		
	T_1–_T_0_	T_2–_T_0_	T_2–_T_1_	T_1–_T_0_	T_2–_T_0_	T_2–_T_1_
Child symptoms						
Mean difference ± SD	1.57 ± 0.47	1.57 ± 0.47	0	1.74 ± 0.66	1.74 ± 0.66	0
* r*	0.87	0.87	N/A	0.93	0.93	N/A
P*	** < 0.001**	** < 0.001**	1	** < 0.001**	** < 0.001**	1
Child function						
Mean difference ± SD	4.34 ± 1.83	4.43 ± 1.80	0.06 ± 0.41	4.04 ± 1.79	4.07 ± 1.74	0.07 ± 0.85
* r*	0.88	0.87	0.22	0.85	0.85	0.05
P*	** < 0.001**	** < 0.001**	0.35	** < 0.001**	** < 0.001**	0.69
Child psychology						
Mean difference ± SD	1.68 ± 1.77	1.71 ± 1.76	0.03 ± 0.17	1.73 ± 1.89	1.72 ± 1.75	0.01 ± 1.12
* r*	0.68	0.68	0.17	0.72	0.71	0.04
P*	** < 0.001**	** < 0.001**	0.16	** < 0.001**	** < 0.001**	0.76
Child self-image and social interaction						
Mean difference ± SD	1.57 ± 1.51	1.55 ± 1.51	0	1.37 ± 1.57	1.21 ± 1.62	0.16 ± 0.99
* r*	0.58	0.58	N/A	0.63	0.61	0.29
P*	** < 0.001**	** < 0.001**	1	** < 0.001**	** < 0.001**	**0.02**
Overall CIS						
Mean difference ± SD	9.16 ± 3.06	9.28 ± 3.02	0.07 ± 0.62	8.88 ± 3.01	8.74 ± 3.01	0.24 ± 1.48
* r*	0.87	0.87	0.15	0.86	0.87	0.16
P*	** < 0.001**	** < 0.001**	0.39	** < 0.001**	** < 0.001**	0.2
Family distress						
Mean difference ± SD	2.91 ± 1.10	2.94 ± 1.09	0.03 ± 0.38	2.28 ± 1.61	0.47 ± 1.67	0.26 ± 1.28
* r*	0.87	0.87	0.17	0.74	0.74	0.16
P*	** < 0.001**	** < 0.001**	0.50	** < 0.001**	** < 0.001**	0.19
Family function						
Mean difference ± SD	1.47 ± 1.46	1.50 ± 1.46	0.03 ± 1.04	1.25 ± 1.48	1.26 ± 1.49	0.01 ± 1.18
* r*	0.87	0.87	0.09	0.82	0.82	0.17
P*	** < 0.001**	** < 0.001**	1	** < 0.001**	** < 0.001**	0.16
Overall FIS						
Mean difference ± SD	3.32 ± 1.89	3.38 ± 1.92	0.06 ± 1.36	3.50 ± 2.15	3.37 ± 2.21	0.16 ± 1.74
* r*	0.87	0.87	N/A	0.84	0.84	0.12
P*	** < 0.001**	** < 0.001**	1	** < 0.001**	** < 0.001**	0.32
Over ECOHIS score						
Mean difference ± SD	13.29 ± 5.08	13.41 ± 5.09	0.12 ± 1.40	12.46 ± 4.83	12.18 ± 4.85	0.28 ± 2.41
* r*	0.87	0.87	0.22	0.87	0.87	0.20
P*	** < 0.001**	** < 0.001**	0.08	** < 0.001**	** < 0.001**	0.10

P*, Wilcoxon signed-rank test; *p*-value was set to 0.05

T_0_, baseline; T_1_, first follow-up period at 6-month; T_2_, second follow-up period at 12-month

*ECOHIS*, early childhood oral health impact scale; *CIS*, child impact scale; *FIS*: family impact scale

*ZC*, zirconia crown; *RCSC*, resin-bonded composite strip crown

Table 6 presents the Poisson regression model to check the risk factors associated with the overall OHRQOL at 6 and 12 months. No significant difference between the restoration type and success rate at T_1_. While at T_2_, parents of children’s teeth restored with RCSC were 3.22 times more likely to report poor OHRQOL than those restored with ZC. Similarly, the RCSC failure rate showed a significant negative impact on the perceived OHRQOL at T_2_ (3.57 times more likely to have poor OHRQOL). ECOHIS overall score at the baseline was significantly associated with the outcome at T_1_ and T_2_.

**Table 6:** Univariate and multiple Poisson regression for effect of independent predictors on the ECOHIS total score at T_1_and T_2_

Predictors	Unadjusted RR (95% *CI*) at T_1_	Unadjusted RR (95% *CI*) at T_1_	Unadjusted RR (95% *CI*) at T_2_	Unadjusted RR (95% *CI*) at T_2_
Gender				
Girls	1.56 (0.74; 2.31)	1.24 (0.97; 1.78)	1.95 (0.91; 2.57)	1.25 (0.41; 3.82)
Boys	1	1	1	1
Parental perception toward his/her child’s oral health at T_0_				
Poor/fair	-	-	2.82**(1.42; 3.58)	2.19*(1.22; 5.76)
Average			1.20 (0.43; 2.35)	1.14 (0.29; 4.44)
Good/excellent			1	1
Restoration type				
RCSC	1.71 (0.68; 3.69)	1.22 (0.25; 3.12)	3.34*(1.66; 9.04)	3.22*(1.79; 8.73)
ZC	1	1	1	1
Restoration retention				
Success	1.66 (0.75; 3.86)	1.32 (0.52; 4.25)	4.53**(1.72; 6.89)	3.57**(1.22; 4.81)
Failure	1	1	1	1
Color match				
Match	-	-	2.14*(1.13; 3.45)	1.96*(1.05; 2.41)
Mismatch			1	1
OHRQOL at T_0_ (quantitative variable)	2.85*(1.54; 3.35)	2.44*(1.42; 3.02)	2.54*(1.24; 3.78)	2.37*(1.18; 3.70)

*RR*, relative risk; *CI*: confidence interval; *p < 0.05 and **p < 0.01

T_1_, First follow-up period at 6-month; T_2_, second follow-up period at 12-month

*ECOHIS*, early childhood oral health impact scale

*ZC*: zirconia crown; *RCSC*: resin-bonded composite strip crown; *dmft*: decayed, missing, filled teeth

## Discussion

The study was designed to investigate the change of OHRQOL after restoring anterior teeth of the young children with two esthetic restorations. The null hypothesis (*H*_*0*_) assumed that ZC and RCSC conferred no diverse parental perception toward their children’s OHRQOL. The prospective nature of the study over 12 months permits a better understanding of the treatment influence on a dynamic process such as OHRQOL. Deep and comprehensive identification of the risk factors associated with the children’s OHRQOL is another benefit of the longitudinal design.

The trial justified the use of Wilson and Cleary conceptual model to study the OHRQOL associated with esthetic restorations because of its efficacy, consistency, and clarity. The model can be used for all ages, health or disease statuses. Wilson and Cleary model deals with varied health aspects and allows subjective perception of the OHRQOL to inspect the causality and interaction of different domains such as bio-physiological domains, environmental and individual characteristics [28].

Parental perception toward their children’s OHRQOL using the ECOHIS instrument proves a good validity and reliability [29]. ECOHIS is a credited tool to assess the change over time of OHRQOL for preschool children in several former studies [27, 30–32]. Parents showed a high response rate and adherence to attend the follow-up appointments. This reflects the parent’s keenness and great concern for keeping their children’s oral health in an adequate status. High responsiveness was also achieved in previously published studies [27, 33].

Several concerns must be taken into consideration before comparing our results with other studies. Some of these concerns can be summarized: (i) the difference in study design, (ii) cultural and social norms of the participants, (iii) sample size, (iv) OHRQOL measure tool, and (v) the method of data acquisition. However, highlighting some results is beneficial. At the baseline, the findings of the current study confirmed the negative impact of ECC on OHRQOL of preschool children and the significant enhancement after treatment regardless of the restoration type. For instance, the frequencies of often/very often responses regarding the toothache and difficulties during eating, drinking, and/or biting were the dominant scores at the baseline. Because of pain and function limitations, parents reported a high rate of absenteeism from work and school. This was consistent with the findings of previous studies [34, 35]. The child self-image/social interaction domain was significantly differing before and after treatment, as well as between the ZC and RCSC. This could be attributed to the higher percentage of enrolled children who were aged from 5 to 6 years. This age group is more self-conscious, aware, and sensitive to the esthetic differences, especially in the anterior teeth. This was agreed with the findings of Soares et al., who confirmed that children with poorly esthetic anterior teeth were 4.69 more likely to perceive inferior social perception scores [36]. On the contrary, our finding was contradicted by the previous research of Sonbol et al., who didn’t reveal any significant association before and after the restoration of anterior teeth with ZCs [37]. The difference in the children’s age and the number of recruited children may explain the controversy. They included much younger than ours, with an average age of 39 ± 5.7 months.

Our results emphasized the significant superiority of the overall CIS of the ZC group over the RCSC group at the two follow-up occasions. This could be attributed to the higher clinical success of ZC at the two follow-ups. The majority of ZCs were retained in situ and showed a significant superior color (at T_1_ and T_2_) and contour (at T_2_) qualities over the RSCS. Regarding the retention of ZC, our findings were similar to the outcomes published by Walia et al. 2014 [38] and comparable to the findings of Alaki et al. 2020 who reported a success rate of 98.3% at 6 and 12 months [13]. Retention rate of THE RCSC in the present trial was consistent with that proposed by Kupietzky et al. (88%) [20] and Ram and Fuks (80%) [10]. The crown reduction for both restorations extended from 1 to 2 mm subgingivally according to the manufacturer’s guidelines. This was to ensure no crown margin exposure, healthy gingival adaptation and maximizes retention. Wear of opposing teeth was obvious in the ZC group at the end of follow-up period. This was in agreement with the findings of Alaki et al. 2020 who reported 7 teeth that suffered from enamel loss after 12 months [13]. Similarly, Walia et al. 2014 found an enamel loss of 4 teeth out of 38 ZCs [38]. Concerning the restoration esthetics, the significant inferiority of RCSC compared to ZC might be because of the pulp therapy which was responsible for the discoloration of the composite resin. However, our color mismatching was much less than that reported by a previous study [20]. This could be attributed to the use of a layer of glass ionomer to separate the composite resin from the underlying pulp capping material and the difference in the treatment modality of the pulp (i.e., we used pulpotomy while Kupietzky et al. adopted and the root canals were filled with an iodoform paste “endoflas” which may be responsible for the yellow discoloration of the composite resin. On the other hand, the ZC provides reasonable esthetic properties with superior translucency with highly polished and glazed surface [39]. The changed contour of the RCSC could be explained on the basis of restoration material loss [40].

Regarding the FIC, the frequency of negative responses of parental upsets and feeling guilty was high before their children received the treatment. Postoperative significant improvements were notified at the follow-ups (T_1_ and T_2_). This reflects the negative impact of ECC on parental distress which could be linked to their children suffering from toothache and chewing troubles. This was in line with Novaes et al. outcomes which consolidated parental perception of guilt with their children’s oral health. They concluded that the severity of the oral condition was directly proportionated with the parental sensation of guilt [27]. Also, a study conducted by Guedes et al. proved the significant association between acquiring of new carious lesions among 352 preschool children who were tracked for 2 years and high family distress scores [41].

Regarding the intragroup difference between the first and second follow-ups (i.e., the difference between T_2_ and T_1_), no significant change was found in the ZC group. This could be explained by the steadiness of the ZC success rate over the follow-ups. Subsequently, children perceived minor changes in the OHRQOL. Similarly, the changes in the RCSC group were not significant except for the child’s self-image/social interaction items. This finding could be attributed to the high rate of falling down of RCSC and color mismatch observed at the end of the follow-up period.

Concerning the secondary outcome of the current study, the regression analysis model determined the risk factors which were associated with the overall OHRQOL change over 6 and 12 months. Restoration loss was significantly linked with inferior OHRQOL. While the effect of this predictor showed no influence on OHRQOL after 6 months. This could be explained by the high loss rate of RCSC at the end of follow-up which confirmed by that OHRQOL perception of children who received RCSC was 3.22 times more likely to be worse than that of children treated with ZC. This was in line with the conclusion of Salami et al., who confirmed the significant parental dissatisfaction with the RCSC durability. They reported that RCSC showed the least retention rate when compared to ZC and PVSSC [12]. Parental perception toward their children’s general oral health at the baseline and color match was significantly associated with the OHRQOL. This confirms the extent to which parents care about the esthetics of their children’s teeth. Our color match effect conflicts with Salami et al., who didn’t confirm such association [12]. The smaller sample size of Salami et al. trial — only 13 children — may be the reason behind this difference.

### Strengths and limitations

Up to our knowledge, it was the prime study that compared the impact of two esthetic restorations on the OHRQOL of preschool children with ECC. The longitudinal nature of the trial allowed a better understanding of the risk factors and their impact on the OHRQOL of children and their families. The assessment was performed in two successive time periods, which permitted dealing with the OHRQOL as a dynamic process that changed. Another good point was adopting a theoretical model to study the impact of different predictors on the OHRQOL. Finally, the proper small size and high responsiveness of the current study increased the reliability of the outcomes. Also, the inter-examiner reliability and ICC were excellent. The main limitations could be summarized: (i) some of the important were not incorporated into the conceptual model such as the sense of coherence (SOC) which may affect the OHRQOL and (ii) the use of a proxy tool to evaluate the OHRQOL. Some authors claimed that parental perception may not be coordinated with their children’s self-perception.

## Conclusions

Within the limitations of the current study, we can conclude that ECC has a significant negative impact on the OHRQOL of preschool children. A remarkable improvement (i.e., large effect size) was recorded after restoring anterior teeth with ZC or RCSC. However, the OHRQOL perceived by preschool children’s parents treated with ZC was significantly better than those treated with RCSC at the follow-ups. Finally, restoration type, color match, and parental perception toward his/her child’s oral health and OHRQOL at the baselines showed a significant impact on the perceived OHRQOL at the end of the follow-up period.

## Supplementary Information

Below is the link to the electronic supplementary material.

Supplementary file1 (PDF 77 KB)
